# Watercress Extract Reduces Experimental Colitis by Modulating Inflammation and Regulating the Gut Microbiota

**DOI:** 10.3390/nu18142369

**Published:** 2026-07-20

**Authors:** Guangyi Shen, Dekun Cheng, Jathya C. Karunathilaka, Jieun Woo, Donglu Li, Jonica L. Wooton, Patricia Jaynes, Tingting Ju, Weicang Wang

**Affiliations:** 1 Department of Food Science, Purdue University, West Lafayette, IN 47907, USA; shen745@purdue.edu (G.S.); cheng766@purdue.edu (D.C.); jsenarat@purdue.edu (J.C.K.); woo89@purdue.edu (J.W.); li5875@purdue.edu (D.L.); 2 Interdepartmental Nutrition Graduate Program, Purdue University, West Lafayette, IN 47907, USA; 3 Department of Animal Science, Purdue University, West Lafayette, IN 47907, USA; jwooton@purdue.edu (J.L.W.); jaynes@purdue.edu (P.J.); ju48@purdue.edu (T.J.)

**Keywords:** watercress, *Nasturtium officinale*, colitis, gut barrier, gut microbiota, phenethyl isothiocyanate

## Abstract

**Background/Objectives:** Watercress (*Nasturtium officinale*), a cruciferous vegetable rich in phenethyl isothiocyanate (PEITC) and bioactive phytochemicals, has demonstrated anti-inflammatory and cytoprotective activities in multiple disease models; however, its effects on colitis remain insufficiently characterized. In the present study, we investigated the effects of watercress extract supplementation in dextran sulfate sodium (DSS)-induced experimental colitis. **Methods:** Male C57BL/6 mice were fed either a standard AIN-93G diet or a diet supplemented with 0.5% (*w*/*w*) watercress extract for 4 weeks, followed by DSS administration to induce acute colitis. Colonic histopathological injury, immune responses, barrier integrity, and gut microbiota composition were evaluated. In addition, the activity of PEITC, a major bioactive constituent of watercress, was examined in lipopolysaccharide (LPS)-stimulated RAW264.7 macrophages in vitro. **Results:** Watercress extract attenuated DSS-induced colon shortening and reduced histopathological injury. The supplementation of watercress extract also decreased colonic immune cell accumulation, suppressed the expression of pro-inflammatory mediators, and preserved intestinal barrier integrity. The *16S rRNA* gene amplicon sequencing further revealed that watercress extract reshaped gut microbial composition, including increased abundance of *Monoglobus* and *Adlercreutzia*, and reduced abundance of microbial taxa such as *Enterococcus* and *Enterorhabdus*. Moreover, PEITC suppressed LPS-induced inflammatory activation in RAW264.7 macrophages by reducing nitric oxide production, inhibiting p38 MAPK phosphorylation, and downregulating multiple inflammatory cytokines and chemokines. **Conclusions:** These findings demonstrate that watercress extract alleviates experimental colitis through attenuating mucosal inflammation, preserving intestinal barrier function, and modulating the gut microbiota, highlighting watercress as a promising dietary strategy for improving gut health and mitigating intestinal inflammation.

## 1. Introduction

Watercress (*Nasturtium officinale*) is a fast-growing aquatic vegetable belonging to the *Brassicaceae* family that has long been incorporated into human diets and traditional medicinal practices. Watercress is distinguished by its high content of bioactive phytochemicals and micronutrients, positioning it as a promising functional food with potential health-promoting properties [1]. Notably, it is a major natural source of phenethyl isothiocyanate (PEITC), a bioactive isothiocyanate generated through myrosinase-mediated hydrolysis of glucosinolate precursors during tissue disruption or digestion. PEITC has attracted increasing attention due to its anti-inflammatory, antioxidant, and chemopreventive activities through modulation of multiple inflammatory signaling pathways [2,3,4,5,6]. In addition to PEITC, watercress contains a variety of glucosinolates, isothiocyanates, flavonoids, vitamins, minerals, and other antioxidant compounds, which collectively contribute to its antioxidant and immunomodulatory potential [7].

Accumulating evidence suggests that watercress exerts beneficial biological effects through both antioxidant and anti-inflammatory mechanisms. In experimental models, watercress extract has been shown to protect against liver, lung, and kidney injury by reducing oxidative stress markers, including malondialdehyde (MDA), nitric oxide (NO), and protein carbonyls, while enhancing endogenous antioxidant defenses such as glutathione peroxidase (GPx), catalase (CAT), and ferric reducing antioxidant power (FRAP) [8,9,10,11]. Similar antioxidant benefits have also been observed in human studies, where watercress supplementation reduced oxidative stress biomarkers in individuals with physical disabilities and asthma patients [12,13]. Beyond its antioxidant activity, watercress has demonstrated anti-inflammatory effects in multiple disease models by reducing lymphocyte and neutrophil infiltration, vascular congestion, edema, and the production of pro-inflammatory mediators, including macrophage inflammatory protein-2 (MIP-2), interleukin-1β (IL-1β), and tumor necrosis factor-α (TNF-α) [14,15,16,17]. Moreover, watercress leaf methanolic extract reduced dextran sulfate sodium (DSS)-induced colonic inflammation in rat [18]. Collectively, these findings suggest that watercress possesses broad antioxidant, anti-inflammatory, and tissue-protective properties, supporting its potential as a functional food for the prevention and management of chronic inflammatory disorders.

Despite the well-documented antioxidant and anti-inflammatory properties of watercress, its effects on gut microbial composition and its potential role in regulating host responses during colonic inflammation remain insufficiently characterized. These knowledge gaps are particularly relevant to inflammatory bowel disease (IBD), a group of chronic gastrointestinal disorders, including ulcerative colitis and Crohn’s disease, that affect millions of individuals worldwide [19]. The pathogenesis of IBD involves complex interactions among immune dysregulation, epithelial barrier dysfunction, and gut microbial dysbiosis [20]. Increasing evidence suggests that disrupted host–microbe interactions contribute to chronic intestinal inflammation and tissue injury [21]. Consequently, dietary strategies capable of simultaneously modulating inflammatory responses, reinforcing epithelial barrier integrity, and reshaping gut microbial ecosystem have gained increasing attention as complementary approaches for IBD management. Given the bioactive composition of watercress and the established actions of its phytochemicals on inflammatory and anti-oxidative pathways, it is important to elucidate whether watercress can protect against colonic inflammation under colitis conditions.

To address these gaps, the present study aimed to investigate the protective effects of watercress extract supplementation in a DSS-induced colitis model using C57BL/6 mice. Specifically, we aimed to determine whether watercress extract attenuates colonic inflammation and tissue injury, preserves intestinal barrier integrity, and modulates gut microbial composition. In addition, we aimed to examine the anti-inflammatory activity of PEITC, a major bioactive compound enriched in watercress, using lipopolysaccharide (LPS)-stimulated macrophages in vitro. We hypothesized that dietary watercress extract supplementation protects against DSS-induced colitis. Understanding of how watercress regulates mucosal inflammation, epithelial barrier function, and the gut microbiota may support its development as a dietary strategy for mitigating colitis and provide mechanistic insight into how cruciferous vegetables influence diet–gut–immune interactions.

## 2. Methods and Materials

### 2.1. Animal Experiments

Animal experiments were conducted under the protocols approved by the Purdue Institutional Animal Care and Use Committee (IACUC, #1123002447) and were performed with the ARRIVE guidelines. Male C57BL/6 mice (6-week-old, *n* = 6 per group, a total of 12 mice) were obtained from the Jackson Laboratory (Bar Harbor, ME, USA) and maintained in ventilated cages under controlled environmental conditions, with a 12 h light/12 h dark cycle. The mice were distributed randomly into experimental groups to receive either an AIN-93G diet (Dyets, Inc., Bethlehem, PA, USA) or a watercress extract powder supplemented AIN-93G throughout a 4-week study period. No animals were excluded from the analyses after group allocation. The sample size was chosen based on our previous studies using DSS-induced colitis or dietary intervention models that demonstrated sufficient sensitivity to detect biologically relevant differences in disease severity and inflammatory markers [22,23]. The watercress extract powder was purchased from Hard Eight Nutrition (Henderson, NV, USA). The nutritional composition of the watercress extract powder is presented in Table S1. Watercress extract was incorporated into the diet at 0.5% (*w*/*w*). This dietary concentration was chosen based on previous studies reporting beneficial biological effects at comparable doses (500–750 mg/kg body weight/day) [12,14,24]. The intake of watercress extract was estimated to be approximately 700 mg/kg body weight/day, assuming a food consumption of 3.5 g diet per 25 g mouse per day. This estimate corresponds to an approximately 57 mg/kg body weight/day based on body surface area normalization [25]. However, individual food intake was not monitored, and therefore the reported dose represents an estimated rather than directly measured exposure. Diets were replaced every 2–3 days throughout the study. After the 4-week dietary pretreatment period, mice in both the control and watercress extract groups received 1.5% DSS (molecular weight 40 kDa; Thermo Scientific Chemicals, Waltham, MA, USA) in their drinking water for 7 days to induce acute colitis. To minimize potential confounding factors, cages from different treatment groups were housed under identical environmental conditions and regularly rotate throughout the study. At the experimental endpoint, mice were euthanized by carbon dioxide inhalation followed by cervical dislocation to ensure death. The abdominal cavity was then opened, and colon tissues were immediately harvested. Colon length was measured, and tissues were either snap-frozen or fixed for subsequent analyses.

### 2.2. Histological Analysis and Tissue Staining

The distal colon tissues were fixed in 10% neutral-buffered formalin and underwent sectioning and subsequent deparaffinization. Hematoxylin and eosin (H&E) staining was performed on tissue sections for colonic morphology evaluation under a light microscope. To assess histopathological damage, we graded tissue changes based on prior protocols [22], which evaluated crypt structural damage, infiltration of inflammatory cells, thickening of the muscle layer, and depletion of goblet cells. To eliminate potential bias, a blinded researcher executed the histological grading, image acquisition, and quantitative analyses of immunohistochemical signals were performed in a blinded manner. For Periodic Acid–Schiff (PAS) staining, deparaffinized sections were incubated with periodic acid and Schiff’s reagent. PAS^+^ goblet cells were checked under a bright-field microscope and quantified using ImageJ software (Version 1.54s, National Institutes of Health, Bethesda, MD, USA) by calculating the number of PAS-positive cells per crypt. Immunohistochemical (IHC) staining was performed using a horseradish peroxidase (HRP)/diaminobenzidine (DAB) Detection IHC Kit (Abcam, Cambridge, UK). Nonspecific binding was blocked using a protein blocking solution, and sections were incubated overnight at 4 °C using CD45 antibody (Cell Signaling Technology, Beverly, MA, USA; #70257; 1:100 dilution). Sections were counterstained with hematoxylin for 1 min. Negative control staining was performed using a rabbit IgG isotype control antibody (Cell Signaling Technology, #3900; 1:100 dilution). Representative negative control images are provided in Figure S1. CD45 staining intensity was quantified using ImageJ software.

### 2.3. Quantitative Polymerase Chain Reaction (qPCR) Analysis

Total RNA was extracted from distal colon tissues or RAW 264.7 macrophages using TRIzol reagent (Ambion, Austin, TX, USA). High-Capacity cDNA Reverse Transcription Kit (Applied Biosystems, Woburn, MA, USA) was applied to synthesize complementary DNA (cDNA). PowerUp SYBR Green Master Mix (Thermo Fisher Scientific, Waltham, MA, USA) was used for qPCR analysis with the CFX96 Detection System (Bio-Rad, Hercules, CA, USA). Mouse-specific primers were ordered from the Thermo Fisher Scientific. Primer specificity was confirmed by melt curve analysis, which demonstrated a single amplification product for each primer pair. Detailed primer information is provided in Table S2. Relative gene expression was normalized using the geometric mean of two reference genes (*glyceraldehyde-3-phosphate dehydrogenase* and *β-actin*) and calculated using the 2^−ΔΔCt^ method. Gene expression analyses were conducted using biological replicates from animal and cell experiments.

### 2.4. Cell Assays

RAW 264.7 macrophages (ATCC, Manassas, VA, USA) were maintained in Dulbecco’s Modified Eagle Medium (DMEM, Corning Inc., Corning, NY, USA) with 10% fetal bovine serum (FBS). RAW264.7 macrophages were stimulated with lipopolysaccharide (LPS, Sigma-Aldrich, St. Louis, MO, USA, cat# L3012) in the presence or absence of phenethyl isothiocyanate (PEITC, Thermo Scientific Chemicals, #265160050), a bioactive compound derived from watercress. NO generation was quantified by measuring nitrite levels in the culture supernatant using the Griess Reagent Nitrite Measurement Kit (Cell Signaling Technology, #13547). For inflammatory gene expression analysis, RAW 264.7 macrophages were pre-treated with PEITC (1 or 3 μM) for 1 h, followed by co-treatment with LPS (10 ng/mL) for 2 h. qPCR analysis was performed as described in Section 2.3.

### 2.5. Western Blotting

RAW 264.7 cells were pre-treated with PEITC (1 or 3 μM) for 1 h and treated together with LPS (10 ng/mL) for 12 h. Cells were then lysed in RIPA lysis buffer (Boston Bioproducts, Ashland, MA, USA, #BP-115DG). The concentration of protein was measured using BCA protein assay kit (Thermo Fisher Scientific, #23227). For protein separation, equivalent protein amounts were loaded on sodium dodecyl sulfate—polyacrylamide gel electrophoresis (SDS–PAGE) gels, followed by transfer to nitrocellulose membranes. The membranes were then incubated with antibodies all obtained from Cell Signaling Technology (MA, USA): p38 antibody (#9212, 1:1000 dilution), phospho-p38 antibody (#4511, 1:1000 dilution), iNOS antibody (#13120, 1:1000 dilution), CD86 antibody (#19589, 1:1000 dilution), and β-actin antibody (#3700, 1:1000 dilution). Following the washing step, membranes were incubated with 800CW goat anti-rabbit IgG (LI-COR Biosciences, Lincoln, NE, USA; #926-32211, 1:10,000 dilution) or 680RD goat anti-mouse IgG (LI-COR Biosciences; #926-68170, 1:10,000 dilution). Western blot analyses were performed using three independent biological replicates (*n* = 3) per treatment group.

### 2.6. Fecal DNA Extraction and 16S rRNA Sequencing

Total genomic DNA was extracted from fecal samples using the Quick-DNA Fecal/Soil Microbe Miniprep Kit (Zymo Research, Tustin, CA, USA) and library preparation and *16S rRNA* gene sequencing were conducted at the Purdue Genomics and Genome Editing Facility using an Illumina MiSeq platform (Illumina, San Diego, CA, USA) as we conducted before [22]. Raw sequencing reads were processed using QIIME2 (version 2024.10) pipeline, including demultiplexing and denoising steps. Alpha diversity metrics were calculated and principal coordinate analysis (PCoA) was performed using RStudio (version 2024.12.1). Microbial taxa with a relative abundance of less than 0.005% across all samples were ex-cluded from downstream analyses and visualizations.

### 2.7. Statistical Analysis

All the data are presented as the mean ± standard error of the mean (SEM). The normality of data distributions was assessed by the Shapiro–Wilk test, and equal variance of data was determined using Levene’s test. Student’s *t* test or Wilcoxon–Mann–Whitney test (when the normality test fails) were used to determine the statistical comparisons between two groups. Statistical comparison of three and more groups was analyzed using one-way ANOVA followed by Tukey’s post hoc test. All these data analyses were performed by using SigmaPlot software (Version 11.0, Grafiti LLC, Palo Alto, CA, USA). For *16S rRNA* gene amplicon sequencing, statistical comparisons of α-diversity indices were performed using Student’s *t* test or Mann–Whitney U test, while permutational multivariate analysis of variance (PERMANOVA, 999 permutations) will be applied to assess differences in β-diversity.

## 3. Results

### 3.1. Watercress Extract Mitigates Disease Severity in Experimental Colitis

**Figure 1:**
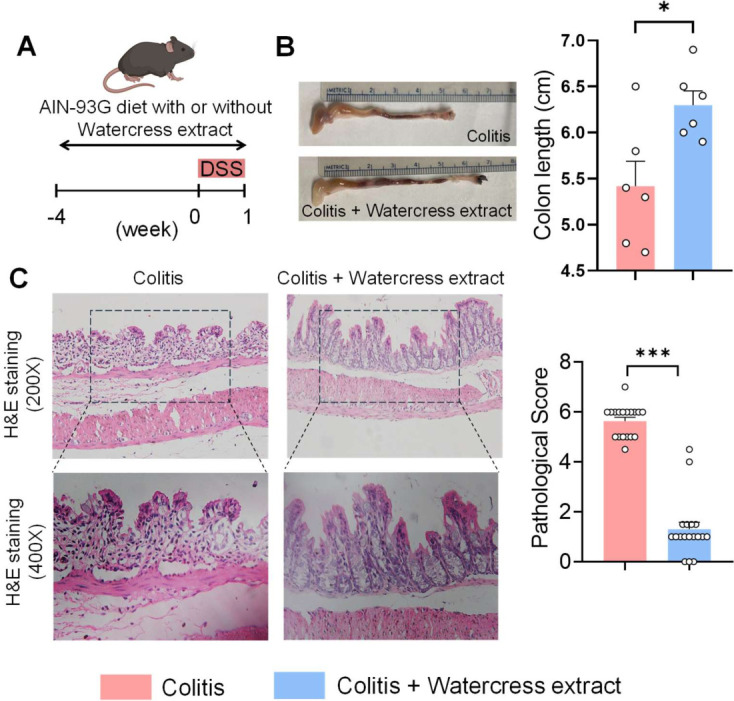
Watercress extract supplementation decreased dextran sulfate sodium (DSS)-induced colitis. (**A**) Scheme of animal experiment. C57BL/6 mice were fed either a standard diet or a watercress extract-containing diet (0.5% *w*/*w*) for 4 weeks, followed by administration of DSS (1.5% *w*/*v*) in drinking water for 7 days to induce acute colitis. (**B**) Colon length measurements. (**C**) Hematoxylin and eosin (H&E) staining (magnification 200× and 400×) and pathological score analysis (*n* = 18 random fields per group). The results are expressed as mean ± SEM. *n* = 6 mice per group. * *p* < 0.05, *** *p* < 0.001.

Mice were fed either an AIN-93G diet or an AIN-93G diet supplemented with watercress extract for 4 weeks, followed by 7 days of DSS administration (Figure 1A). The watercress extract powder was administered at 0.5% (*w*/*w*) in the diet, a dose selected based on prior studies reporting beneficial effects of watercress extract at similar exposure levels (500–750 mg/kg/day) [12,14,24]. During the dietary conditioning period, watercress extract supplementation did not result in detectable changes in body weight compared with the control group (Figure S2). Following DSS challenge, mice receiving watercress extract showed attenuated colon shortening compared with DSS-treated mice fed the control diet, suggesting reduced severity of colonic inflammation and tissue injury (Figure 1B). Consistent with these observations, colonic pathological alterations, including epithelial erosion, crypt architectural disruption, and pronounced inflammatory cell infiltration, were reduced in mice supplemented with watercress extract (Figure 1C), indicating preservation of mucosal integrity and reduced inflammatory damage. In addition, watercress extract supplementation did not significantly affect body weight, liver weight, or spleen weight under colitis conditions (Figure S3). Collectively, these findings demonstrate that dietary supplementation with watercress extract alleviates disease severity and histological injury in DSS-induced colitis, supporting a protective role of watercress against acute colonic inflammation.

### 3.2. Watercress Extract Consumption Reduced the Pro-Inflammatory Response During Colitis

**Figure 2:**
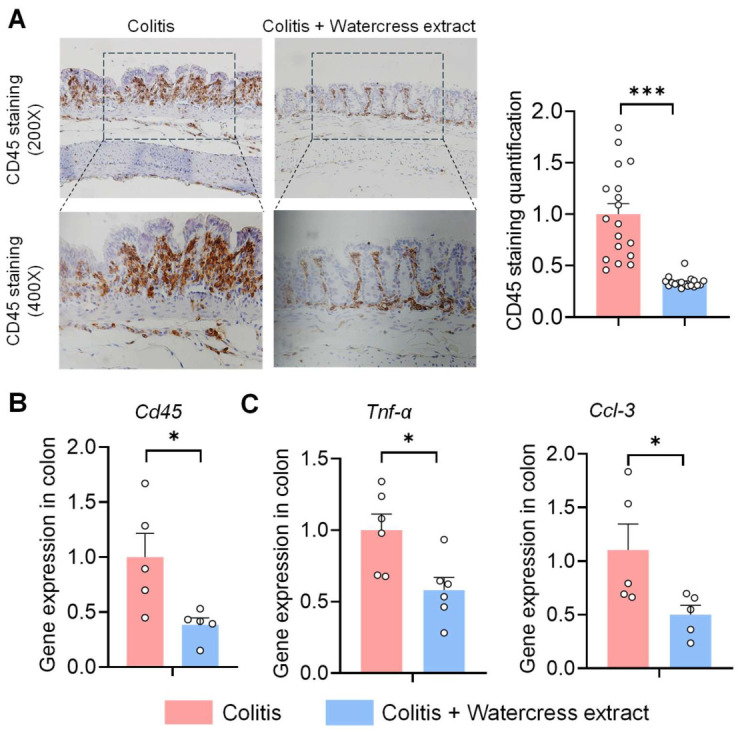
Watercress extract consumption limited immune cell infiltration and pro-inflammatory cytokine expression during colitis. (**A**) Immunohistochemical staining of CD45 (magnification 200× and 400×) and quantification of CD45 staining intensity in colon (*n* = 18 random fields per group). (**B**) Gene expression of *Cd45* in colon. (**C**) Gene expression of *Tnf-α* and *Ccl-3* in colon. The results are expressed as mean ± SEM. *n* = 5–6 mice per group. * *p* < 0.05, *** *p* < 0.001.

We next investigated whether watercress extract consumption modulates colonic immune activation during DSS-induced colitis. Immunohistochemical staining revealed a reduction in CD45^+^ immune cell accumulation in the colonic mucosa of watercress extract–supplemented mice compared with DSS-treated controls (Figure 2A), indicating decreased leukocyte infiltration into inflamed colonic tissue. Consistent with these histological observations, watercress extract supplementation reduced the expression of the pan-leukocyte marker *Cd45* in the colons of colitic mice (Figure 2B). In parallel with the reduced immune cell presence, watercress extract supplementation suppressed the expression of key pro-inflammatory mediators, including *Tnf-α* and *C-C motif chemokine ligand 3* (*Ccl3*), which are known to contribute to immune cell recruitment, inflammatory amplification, and leukocyte chemotaxis during experimental colitis (Figure 2C). Together, these findings demonstrate that watercress extract consumption attenuates colonic inflammatory responses during DSS-induced colitis.

### 3.3. Watercress Extract Alleviated Colitis-Related Colonic Barrier Dysfunction

**Figure 3:**
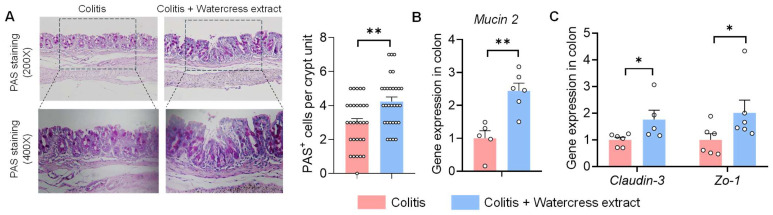
Watercress extract alleviated intestinal barrier dysfunction. (**A**) Periodic acid–Schiff (PAS) staining in colon (magnification 200× and 400×) and quantification of PAS^+^ cells per crypt unit (*n* = 30 random crypt units per group). (**B**) Gene expression of *Mucin 2* in colon. (**C**) Gene expression of *Claudin-3* and *Zo-1* in colon. The results are expressed as mean ± SEM. *n* = 5–6 mice per group. * *p* < 0.05, ** *p* < 0.01.

Disruption of the epithelial barrier is a hallmark of colitis and is commonly associated with goblet cell depletion, impaired mucus production, and destabilization of epithelial junctional complexes. To evaluate whether watercress extract supplementation influences colonic barrier integrity during DSS-induced colitis, we examined mucus-producing goblet cells and the expression of barrier-associated genes in colonic tissues. Periodic acid–Schiff (PAS) staining showing that mice exposed to DSS displayed a pronounced loss of PAS^+^ goblet cells in the colonic epithelium, whereas supplementation with watercress extract increased goblet cell numbers, indicating improved mucus layer maintenance under colitis conditions (Figure 3A). Consistent with the histological observations, gene expression of *Mucin 2*, the primary gel-forming mucin responsible for colonic mucus barrier formation, was elevated in watercress extract-treated colitis mice relative to DSS-only controls (Figure 3B). We further assessed the expression of epithelial junctional components involved in maintaining intestinal permeability. Watercress extract supplementation upregulated the expression of the tight junction components *claudin-3* and *zonula occludens-1* (*Zo-1*), both of which are critical for preserving epithelial junctional organization and limiting paracellular permeability (Figure 3C). Together, these findings demonstrate that watercress supplementation preserves mucus barrier function and reinforces epithelial junctional integrity, thereby mitigating DSS-induced intestinal barrier dysfunction during colitis.

### 3.4. Watercress Extract Consumption Reshaped the Gut Microbial Community

**Figure 4:**
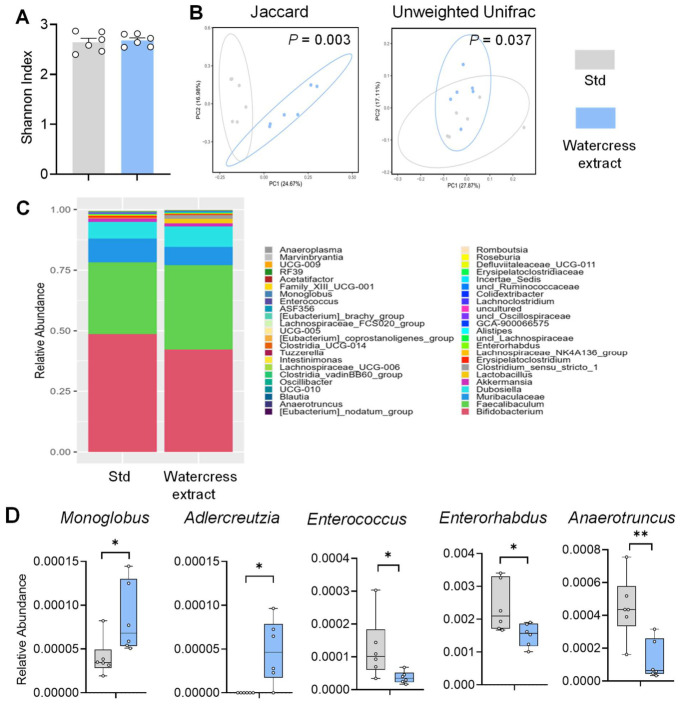
Watercress extract consumption reshaped gut microbial community. Effect of watercress on (**A**) α diversity and (**B**) β diversity of fecal microbiota. (**C**) Effect of watercress on composition of the microbiota at genus levels. (**D**) Relative abundance of representative bacterial genera altered by watercress extract consumption. The results are expressed as mean ± SEM. *n* = 6 mice per group. * *p* < 0.05, ** *p* < 0.01.

To determine whether watercress extract consumption alters the gut microbial system and contributes to protection against colitis, fecal samples were collected from mice receiving either a standard AIN-93G diet or a watercress extract-supplemented diet prior to DSS challenge and were subjected to *16S rRNA* gene amplicon sequencing analysis. Watercress extract consumption did not significantly alter microbial α-diversity indices; however, β-diversity analyses based on Jaccard and unweighted UniFrac distances revealed a clear separation between dietary groups, indicating that watercress supplementation reshaped microbial community structure (Figure 4A,B). Consistent with this compositional shift, taxonomic profiling demonstrated changes in the relative abundance of several bacterial taxa at the phylum and family levels (Figure S4), as well as at the genus level (Figure 4C, Table S3). Specifically, watercress extract supplementation increased the relative abundance of genera including *Monoglobus* and *Adlercreutzia*, while reducing the abundance of genera such as *Enterococcus*, *Enterorhabdus*, and *Anaerotruncus* compared with control-fed mice (Figure 4D,E). Together, these findings demonstrate that watercress extract intake reprograms the gut microbial ecosystem prior to inflammatory challenge, favoring a microbiota profile associated with intestinal homeostasis and potentially preconditioning the host for enhanced protection against DSS-induced colitis.

### 3.5. Phenethyl Isothiocyanate (PEITC), a Bioactive Compound Enriched in Watercress Extract, Attenuated LPS-Induced Inflammatory Signaling In Vitro

**Figure 5:**
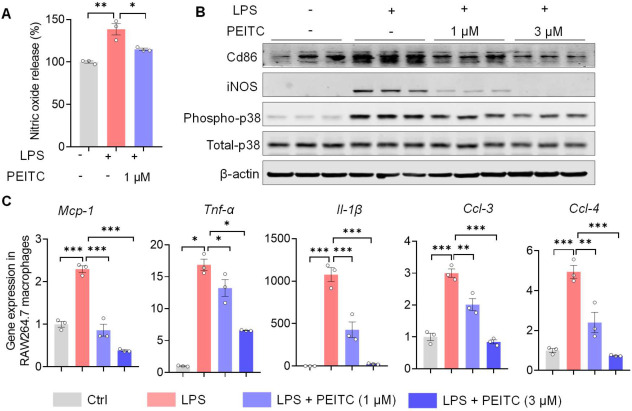
Phenethyl isothiocyanate (PEITC), a bioactive compound enriched in watercress extract, suppresses inflammatory responses in RAW264.7 macrophages. (**A**) Nitric oxide production in RAW264.7 macrophages. (**B**) Western blot analysis of inflammatory signaling and macrophage activation markers in RAW264.7 macrophages. (**C**) Quantitative PCR analysis of pro-inflammatory cytokine and chemokine gene expression in RAW264.7 macrophages. The data are mean ± SEM. *n* = 3 biological replicates per group. * *p* < 0.05, ** *p* < 0.01, *** *p* < 0.001.

Watercress is a rich dietary source of PEITC, a sulfur-containing phytochemical generated through glucosinolate hydrolysis. To further investigate the molecular mechanisms underlying the anti-inflammatory activity of watercress extract, we evaluated the effects of PEITC in LPS-stimulated RA W264.7 macrophages. PEITC treatment reduced LPS-induced nitric oxide production, indicating suppression of oxidative responses in activated macrophages (Figure 5A). Consistent with these findings, Western blot analysis demonstrated that PEITC treatment dose-dependently reduced the protein level of macrophage activation marker CD86 and inducible nitric oxide synthase (iNOS) (Figure 5B). Notably, PEITC markedly suppressed LPS-stimulated phospho-p38 levels without altering total p38 expression, indicating selective inhibition of inflammatory signaling through the p38 MAPK pathway (Figure 5B). Consistently, PEITC treatment downregulated the expression of multiple pro-inflammatory cytokine and chemokine genes, including *Mcp-1*, *Tnf-α*, *Il-1β*, *Ccl-3*, and *Ccl-4*, in LPS-stimulated RAW264.7 macrophages (Figure 5C). Collectively, these findings demonstrate that PEITC, a major bioactive constituent of watercress, suppresses macrophage inflammatory activation by reducing nitric oxide production, inhibiting p38 MAPK signaling, and downregulating pro-inflammatory cytokine and chemokine expression.

## 4. Discussion

Inflammatory bowel disease (IBD) is an increasingly prevalent global health disorder that affects millions of individuals worldwide and imposes substantial clinical and socioeconomic burdens [26]. Dietary interventions have gained growing attention as promising preventive strategies for IBD because of their abilities to modulate both gut microbial composition and the intestinal inflammatory microenvironment [27]. Among dietary phytochemicals, watercress and its bioactive constituents have recently attracted considerable interest due to their antioxidant, anti-inflammatory, and cytoprotective properties. However, the protective effects of watercress against colitis and the underlying mechanisms remain insufficiently characterized. In the present study, we demonstrated that watercress extract supplementation alleviated colonic inflammation and tissue injury in a DSS-induced colitis mouse model. In addition, watercress extract supplementation attenuated immune cell accumulation and improved gut barrier integrity, suggesting beneficial effects on both mucosal inflammation and epithelial homeostasis. Watercress extract also favorably modulated gut microbial ecosystem by enriching selective bacterial taxa. Furthermore, PEITC, a major bioactive compound enriched in watercress extract, suppressed LPS-induced inflammatory responses in macrophages in vitro, providing mechanistic insight into the anti-inflammatory activity of watercress. Collectively, these findings suggest that watercress may represent a promising dietary approach for alleviating IBD-associated inflammation and supporting gut health.

Colonic tissue injury and barrier dysfunction are key pathological features of colitis and contribute to disease severity. In the present study, dietary watercress extract consumption attenuated colonic injury and dysfunction in an experimental colitis mouse model. Notably, watercress extract supplementation reduced DSS-induced colon shortening and improved histopathological features, including epithelial damage and crypt disruption, indicating an overall reduction in disease severity. Moreover, watercress extract preserved goblet cell abundance and increased the expression of barrier-associated genes, including *Zo-1*, and *Claudin-3*, suggesting improved mucus layer integrity and epithelial junctional stability during colitis. These findings are consistent with previous studies reporting tissue-protective effects of watercress extract in multiple experimental models. Indeed, watercress extract has been shown to reduce ear edema in an irritant contact dermatitis model, attenuate alveolar injury in an asthma model, and inhibit collagen deposition in a lung fibrosis model [9,10,15]. Watercress extract has also been reported to reduce swelling and edema in formalin-induced paw inflammation and protect against gentamicin-induced nephrotoxicity and pathological kidney injury [14,16]. In addition, watercress supplementation has been shown to reduce DNA damage in colonic cells, further supporting its potential protective effects in the intestinal environment [28]. Together, these findings suggest that watercress extract may confer broad tissue-protective effects by limiting inflammatory injury, preserving epithelial integrity, and reducing cellular damage.

Infiltration of immune cells into the intestinal mucosa is a key pathological feature of colitis-associated inflammation [29]. In the present study, watercress extract consumption reduced colitis-associated immune cell infiltration and accumulation in the colon, suggesting that suppression of mucosal immune activation may contribute to its protective effects. In addition, watercress extract supplementation decreased the expression of key inflammatory cytokines and chemokines, including *Tnf-α* and *Ccl3*. The downregulation of these inflammatory mediators indicates that watercress extract may attenuate signaling pathways involved in mucosal immune activation and inflammatory amplification. Consistent with our findings, previous studies have reported that watercress extract reduced lymphocyte and neutrophil infiltration, vascular congestion, and edema in a formalin-induced rat paw edema model [14]. Similarly, watercress extract decreased inflammatory cell infiltration and reduced pro-inflammatory mediators, including MIP-2 and IL-1β, in a croton oil-induced irritant contact dermatitis model [15]. Watercress extract has also been shown to suppress IL-1β in carrageenan-induced paw edema, reduce TNF-α in gentamicin-induced kidney injury, and decrease both TNF-α and IL-1β in carbon tetrachloride-induced hepatotoxicity [14,16,17]. Importantly, human evidence also suggests that watercress consumption can downregulate exercise-induced pro-inflammatory cytokines, including IL-6 and TNF-α, during the recovery phase [30]. Together, these studies support that watercress extract mitigates colonic inflammation by limiting immune cell infiltration and suppressing cytokine and chemokine expression.

The gut microbiome plays a critical role in maintaining intestinal homeostasis and modulating disease initiation and progression. Increasing evidence suggests that dietary modulation of the gut microbiota represents a promising strategy for preventing or alleviating intestinal inflammatory disorders [31,32,33,34]. In the present study, watercress extract consumption markedly altered gut microbial composition prior to DSS challenge. Specifically, the fecal microbiota of watercress-fed mice exhibited enrichment of selective bacterial genera, including *Monoglobus* and *Adlercreutzia*, together with reduced abundance of taxa such as *Enterococcus* and *Enterorhabdus*. Notably, *Monoglobus* has been associated with dietary fiber degradation and carbohydrate fermentation [35], whereas *Adlercreutzia* is linked to beneficial microbial metabolism and maintenance of gut homeostasis [36]. In contrast, elevated abundance of *Enterococcus* and *Enterorhabdus* has frequently been associated with intestinal inflammation, epithelial dysfunction, and microbial dysbiosis [37,38,39,40]. These shifts in microbial composition suggest that watercress consumption may support gut health by promoting beneficial microbial populations while suppressing potentially pathogenic or inflammation-associated taxa. Together, these findings suggest that watercress extract consumption promotes a gut microbial profile associated with improved intestinal homeostasis and reduced inflammatory potential.

Watercress is recognized as a rich dietary source of PEITC. In the present study, our cellular assays identified PEITC as a potential mediator of the anti-inflammatory effects of watercress extract. PEITC suppressed LPS-induced macrophage activation, as shown by reduced M1 macrophage marker CD86, inhibition of p38 MAPK phosphorylation, and downregulation of multiple inflammatory mediators, including *Mcp-1*, *Tnf-α*, *Il-1β*, *Ccl-3*, and *Ccl-4*. Moreover, PEITC attenuated oxidative stress by reducing NO production and decreasing the expression of iNOS, the major enzyme responsible for inducible NO generation during inflammation. Consistent with our findings, previous studies have reported that PEITC suppresses LPS-induced interferon-inducible protein-10 (IP-10), iNOS, and COX-2 expression in RAW264.7 macrophages [2,3]. Moreover, PEITC has been shown to inhibit multiple inflammation-related signaling pathways, including NF-κB activation and RANKL-induced p38 MAPK signaling in macrophages, further supporting its anti-inflammatory activity [3,4]. Beyond macrophages, however, the effects of watercress extract and watercress-derived bioactive compounds on other immune cell populations and cytokine profiles during colitis remain largely unclear. Previous studies have shown that PEITC treatment can enhance anti-tumor T-cell activity and improve anti-cancer immune responses, suggesting broader immunomodulatory functions beyond macrophage regulation [5]. Additional studies should also characterize the effects of watercress and its bioactive compounds on intestinal immune cell populations, including T cells, dendritic cells, and neutrophils, during inflammatory bowel disease progression. Moreover, PEITC treatment has been reported to reduce tumor incidence and multiplicity in colitis-associated carcinogenesis, potentially by attenuating inflammation-associated crypt damage [6]. Therefore, future studies are needed to determine whether PEITC directly contributes to the protective effects of watercress extract against colitis and colitis-associated disorders. Several limitations of this study should be acknowledged. First, although the sample size (*n* = 6 per group) was consistent with previous DSS-induced colitis or dietary intervention studies [22,23], a formal a priori statistical power analysis was not performed. Therefore, future studies incorporating larger cohorts and formal statistical power calculations are warranted to further validate and extend the protective effects of watercress extract. Second, the intake of watercress extract was estimated based on dietary formulation and average food consumption rather than direct measurement of individual intake. As a result, actual exposure levels and inter-animal variability in consumption could not be precisely determined. Future studies incorporating individual food intake measurements will be necessary to better characterize watercress extract exposure and establish dose–response relationships. Third, the estimated dose used in this study corresponds to a human-equivalent dose that may exceed typical dietary intake of watercress, which may limit the direct translation of these findings to habitual human consumption patterns. Finally, although the present study demonstrated beneficial effects of watercress extract on DSS-induced colitis, the specific bioactive compounds responsible for these effects were not comprehensively characterized. Additional phytochemical analyses and mechanistic studies are needed to identify the active constituents and elucidate their individual contributions to the anti-inflammatory and anti-colitic activities of watercress extract. Collectively, these findings provide mechanistic insight into how watercress-derived PEITC may contribute to the anti-inflammatory and protective effects of watercress observed during experimental colitis.

## 5. Conclusions

The present study demonstrates that dietary watercress extract supplementation alleviates DSS-induced colitis by reducing colonic inflammation, limiting tissue injury, and preserving intestinal barrier integrity. Moreover, watercress supplementation promoted a gut microbial profile associated with intestinal homeostasis, characterized by enrichment of potentially beneficial bacterial taxa and reduction in inflammation-associated microbes. Mechanistically, our findings identified PEITC, a major bioactive compound enriched in watercress, as a potential mediator underlying these protective effects. Although these findings provide important evidence supporting the protective effects of watercress against experimental colitis, further investigations using chronic colitis models and human clinical trials are warranted to validate the translational potential of watercress and its bioactive compounds for inflammatory bowel disease prevention and management. Beyond PEITC, watercress contains multiple additional phytochemicals and nutrients that may also contribute to its anti-inflammatory and gut-protective effects. Furthermore, previous studies have suggested that urea derivatives from watercress may function as a natural soluble epoxide hydrolase inhibitor, a pro-inflammatory enzyme involved in colonic inflammation and barrier dysfunction [41,42,43], indicating that additional bioactive mechanisms beyond PEITC may be involved in regulating intestinal inflammation and barrier function. Future studies should also identify other active compounds in watercress and define their individual or synergistic contributions to colitis protection and gut microbial modulation. Collectively, this study expands the functional relevance of watercress in gut health and provides new mechanistic insight into how cruciferous vegetable-derived phytochemicals influence diet–gut–immune interactions. These findings could provide a foundation for the future development of watercress-based dietary interventions for intestinal inflammatory disorders.

## Data Availability

All data generated or analyzed during this study are included in this published article and its Supplementary Materials. The datasets generated during the current study are available from the corresponding author upon reasonable request.

## Supplementary Materials

The following supporting information can be downloaded at: https://www.mdpi.com/article/10.3390/nu18142369/s1, Figure S1. Negative control staining for immunohistochemistry. Representative images of rabbit IgG isotype control staining in colonic tissues from DSS-induced colitis mice with or without watercress extract supplementation (magnification 200×). Figure S2. Watercress extract supplementation did not affect body weight during the dietary pretreatment period. Six-week-old male C57BL/6 mice were fed either a standard AIN-93G diet (Std) or a modified AIN-93G diet supplemented with 0.5% (*w*/*w*) watercress extract for 4 weeks. Data are presented as mean ± SEM. *n* = 6 mice per group. No significant difference was detected between groups. Figure S3. Watercress extract supplementation did not influence overall body weight loss, spleen weight, or liver weight during DSS-induced colitis. (A) Body weight changes during DSS treatment. (B) Spleen weight and (C) liver weight at the experimental endpoint. Data are presented as mean ± SEM. *n* = 6 mice per group. No significant differences were detected between groups. Figure S4. Watercress extract supplementation alters gut microbial composition at phylum and family levels. Fecal samples collected from mice fed either a standard AIN-93G diet (Std) or a watercress extract-supplemented diet for 4 weeks prior to DSS administration were subjected to 16S rRNA gene amplicon sequencing analysis. Relative taxonomic composition of the fecal microbiota is shown at (A) phylum level and (B) family level. Table S1. Nutritional composition of the watercress extract powder. Table S2. Sequences of primers in qPCR analysis. Table S3. Effects of watercress extract on the composition of gut microbiota at genus levels (results are expressed as relative abundance).

## References

[1] Maluwa C., Zinan’dala B., Chuljerm H., Parklak W., Kulprachakarn K. (2025). Watercress (*Nasturtium officinale*) as a Functional Food for Non-Communicable Diseases Prevention and Management: A Narrative Review. Life.

[2] Park H.J., Kim S.J., Park S.J., Eom S.H., Gu G.J., Kim S.H., Youn H.S. (2013). Phenethyl isothiocyanate regulates inflammation through suppression of the TRIF-dependent signaling pathway of Toll-like receptors. Life Sci..

[3] Rose P., Won Y.K., Ong C.N., Whiteman M. (2005). Beta-phenylethyl and 8-methylsulphinyloctyl isothiocyanates, constituents of watercress, suppress LPS induced production of nitric oxide and prostaglandin E2 in RAW 264.7 macrophages. Nitric Oxide.

[4] Murakami A., Song M., Ohigashi H. (2007). Phenethyl isothiocyanate suppresses receptor activator of NF-kappaB ligand (RANKL)-induced osteoclastogenesis by blocking activation of ERK1/2 and p38 MAPK in RAW264.7 macrophages. Biofactors.

[5] Lin J.R., Liao W.C., Chu Y.H., Chou Y.C., Liu C.H. (2025). Phenethyl isothiocyanate modulates macrophage migration inhibitory factor and suppresses malignant phenotypes of glioblastoma cells. Food Funct..

[6] Cheung K.L., Khor T.O., Huang M.T., Kong A.N. (2010). Differential in vivo mechanism of chemoprevention of tumor formation in azoxymethane/dextran sodium sulfate mice by PEITC and DBM. Carcinogenesis.

[7] Klimek-Szczykutowicz M., Szopa A., Ekiert H. (2018). Chemical composition, traditional and professional use in medicine, application in environmental protection, position in food and cosmetics industries, and biotechnological studies of *Nasturtium officinale* (watercress)—A review. Fitoterapia.

[8] Azarmehr N., Afshar P., Moradi M., Sadeghi H., Sadeghi H., Alipoor B., Khalvati B., Barmoudeh Z., Abbaszadeh-Goudarzi K., Doustimotlagh A.H. (2019). Hepatoprotective and antioxidant activity of watercress extract on acetaminophen-induced hepatotoxicity in rats. Heliyon.

[9] Shakerinasab N., Bejeshk M.A., Pourghadamyari H., Najafipour H., Eftekhari M., Mottaghipisheh J., Omidifar N., Azizi M., Rajizadeh M.A., Doustimotlagh A.H. (2022). The Hydroalcoholic Extract of *Nasturtium officinale* Reduces Lung Inflammation and Oxidative Stress in an Ovalbumin-Induced Rat Model of Asthma. Evid. Based Complement. Altern. Med..

[10] Ramezani S., Javadi I., Kokhdan E.P., Omidifar N., Nikbakht J., Sadeghi H., Doustimotlagh A.H., Danaei N., Abbasi R., Sadeghi H. (2021). Protective and therapeutic effects of ethanolic extract of *Nasturtium officinale* (watercress) and vitamin E against bleomycin-induced pulmonary fibrosis in rats. Res. Pharm. Sci..

[11] Doustimotlagh A.H., Kokhdan E.P., Vakilpour H., Khalvati B., Barmak M.J., Sadeghi H., Asfaram A. (2020). Protective effect of *Nasturtium officinale* R. Br and quercetin against cyclophosphamide-induced hepatotoxicity in rats. Mol. Biol. Rep..

[12] Clemente M., Miguel M.D., Felipe K.B., Gribner C., Moura P.F., Rigoni A.R.R., Parisotto E.B., Piltz M.T., Valdameri G., Henneberg R. (2020). Biomarkers of oxidative stress and inflammation in people witha physical disability treated with a standardized extract of *Nasturtium officinale*: A randomized, double-blind, and placebo-controlled trial. Phytother. Res..

[13] Shakerinasab N., Mottaghipisheh J., Eftekhari M., Sadeghi H., Bazarganipour F., Abbasi R., Doustimotlagh A.H., Iriti M. (2024). The hydroalcoholic extract of *Nasturtium officinale* reduces oxidative stress markers and increases total antioxidant capacity in patients with asthma. J. Ethnopharmacol..

[14] Mostafazadeh M., Sadeghi H., Sadeghi H., Zarezade V., Hadinia A., Panahi Kokhdan E. (2022). Further evidence to support acute and chronic anti-inflammatory effects of *Nasturtium officinale*. Res. Pharm. Sci..

[15] Camponogara C., Silva C.R., Brusco I., Piana M., Faccin H., de Carvalho L.M., Schuch A., Trevisan G., Oliveira S.M. (2019). *Nasturtium officinale* R. Br. effectively reduces the skin inflammation induced by croton oil via glucocorticoid receptor-dependent and NF-kappaB pathways without causing toxicological effects in mice. J. Ethnopharmacol..

[16] Shahani S., Behzadfar F., Jahani D., Ghasemi M., Shaki F. (2017). Antioxidant and anti-inflammatory effects of *Nasturtium officinale* involved in attenuation of gentamicin-induced nephrotoxicity. Toxicol. Mech. Methods.

[17] Soudkhah S., Keyghobadi S., Shadboorestan A., Gholami M., Omidi Sarajar B., Salek Maghsoudi A., Omidi M., Mohammadi Motamed S., Akbarzadeh Kolahi S., Rastegar-Pouyani N. (2025). The hydroalcoholic extract of *Nasturtium officinale* protectively inhibits apoptotic and inflammatory pathways in hepato- and nephrotoxicity: An in vivo study. Avicenna J. Phytomed..

[18] Mahmoud M.H., Mjery Y., Fatani S.H., Jan A., Taymour S., Abdelsalam K., Nasif W.A., Mukhtar M.H., Eldein M.M.N. (2026). Protective effects of watercress (*Nasturtium officinale*) leaf methanolic extract against dextran sodium sulfate induced colon inflammation in rats. Inflammopharmacology.

[19] Yang K., Zhang C., Gong R., Jiang W., Ding Y., Yu Y., Chen J., Zhu M., Zuo J., Huang X. (2025). From west to east: Dissecting the global shift in inflammatory bowel disease burden and projecting future scenarios. BMC Public Health.

[20] Zhang Y.Z., Li Y.Y. (2014). Inflammatory bowel disease: Pathogenesis. World J. Gastroenterol..

[21] Abraham C., Medzhitov R. (2011). Interactions between the host innate immune system and microbes in inflammatory bowel disease. Gastroenterology.

[22] Woo J., Cheng D., Long E.A., Whitney K.L., Shen G., Reddivari L., Jiang Q., Simsek S., Ju T., Wang W. (2026). Hemp seed mitigates colonic inflammation through macrophage polarization and microbiota-barrier axis restoration. Food Funct..

[23] Cheng D., Shen G., Pathiranage J.C., Zhao Y., Xie J., Wang Q., Ferreira C.R., Zheng Q., Yuan C., Wang W. (2026). Perfluorooctanoic acid exposure disrupts gut microbiota and aggravates experimental colitis. Environ. Pollut..

[24] Sadeghi H., Mostafazadeh M., Sadeghi H., Naderian M., Barmak M.J., Talebianpoor M.S., Mehraban F. (2014). In vivo anti-inflammatory properties of aerial parts of *Nasturtium officinale*. Pharm. Biol..

[25] Reagan-Shaw S., Nihal M., Ahmad N. (2008). Dose translation from animal to human studies revisited. FASEB J..

[26] Ruan G., Sun Y., Yu Z., Bai X., Yang H., Qian J. (2025). Global, regional, and national burden of inflammatory bowel disease from 1990 to 2021: Findings from the Global Burden of Disease 2021. Gastroenterol. Rep..

[27] Godala M., Gaszynska E., Zatorski H., Malecka-Wojciesko E. (2022). Dietary Interventions in Inflammatory Bowel Disease. Nutrients.

[28] Boyd L.A., McCann M.J., Hashim Y., Bennett R.N., Gill C.I., Rowland I.R. (2006). Assessment of the anti-genotoxic, anti-proliferative, and anti-metastatic potential of crude watercress extract in human colon cancer cells. Nutr. Cancer.

[29] Zhang M., Li X., Zhang Q., Yang J., Liu G. (2023). Roles of macrophages on ulcerative colitis and colitis-associated colorectal cancer. Front. Immunol..

[30] Schulze H., Hornbacher J., Wasserfurth P., Reichel T., Gunther T., Krings U., Kruger K., Hahn A., Papenbrock J., Schuchardt J.P. (2021). Immunomodulating Effect of the Consumption of Watercress (*Nasturtium officinale*) on Exercise-Induced Inflammation in Humans. Foods.

[31] Matson V., Gajewski T.F. (2022). Dietary modulation of the gut microbiome as an immunoregulatory intervention. Cancer Cell.

[32] Fernandez-Ruiz I. (2021). Modulating the gut microbiota with dietary interventions to protect against cardiometabolic disease. Nat. Rev. Cardiol..

[33] Dasriya V.L., Samtiya M., Ranveer S., Dhillon H.S., Devi N., Sharma V., Nikam P., Puniya M., Chaudhary P., Chaudhary V. (2024). Modulation of gut-microbiota through probiotics and dietary interventions to improve host health. J. Sci. Food Agric..

[34] Ayten S., Bilici S. (2024). Modulation of Gut Microbiota Through Dietary Intervention in Neuroinflammation and Alzheimer’s and Parkinson’s Diseases. Curr. Nutr. Rep..

[35] Kim C.C., Lunken G.R., Kelly W.J., Patchett M.L., Jordens Z., Tannock G.W., Sims I.M., Bell T.J., Hedderley D., Henrissat B. (2019). Genomic insights from *Monoglobus pectinilyticus*: A pectin-degrading specialist bacterium in the human colon. ISME J..

[36] Zhang Z., Yang Z., Lin S., Jiang S., Zhou X., Li J., Lu W., Zhang J. (2025). Probiotic-induced enrichment of *Adlercreutzia equolifaciens* increases gut microbiome wellness index and maps to lower host blood glucose levels. Gut Microbes.

[37] Zhou Y., Chen H., He H., Du Y., Hu J., Li Y., Li Y., Zhou Y., Wang H., Chen Y. (2016). Increased Enterococcus faecalis infection is associated with clinically active Crohn disease. Medicine.

[38] Sun Y., Huang X., Zhang Y., Bao W., Lu Z., Zhao W., Rukeya Y., He P., Qi J., Liu S. (2025). Enterococcus faecalis hijacks FABP2 to activate quorum-sensing signals and aggravate Crohn’s disease by inducing gut dysbiosis. Gut.

[39] Balish E., Warner T. (2002). Enterococcus faecalis induces inflammatory bowel disease in interleukin-10 knockout mice. Am. J. Pathol..

[40] Clavel T., Duck W., Charrier C., Wenning M., Elson C., Haller D. (2010). *Enterorhabdus caecimuris* sp. nov., a member of the family Coriobacteriaceae isolated from a mouse model of spontaneous colitis, and emended description of the genus Enterorhabdus Clavel et al. 2009. Int. J. Syst. Evol. Microbiol..

[41] Kitamura S., Morisseau C., Harris T.R., Inceoglu B., Hammock B.D. (2017). Occurrence of urea-based soluble epoxide hydrolase inhibitors from the plants in the order Brassicales. PLoS ONE.

[42] Wang W., Yang J., Zhang J., Wang Y., Hwang S.H., Qi W., Wan D., Kim D., Sun J., Sanidad K.Z. (2018). Lipidomic profiling reveals soluble epoxide hydrolase as a therapeutic target of obesity-induced colonic inflammation. Proc. Natl. Acad. Sci. USA.

[43] Wang Y., Yang J., Wang W., Sanidad K.Z., Cinelli M.A., Wan D., Hwang S.H., Kim D., Lee K.S.S., Xiao H. (2020). Soluble epoxide hydrolase is an endogenous regulator of obesity-induced intestinal barrier dysfunction and bacterial translocation. Proc. Natl. Acad. Sci. USA.

