# Supplementary material for: Watercress Extract Reduces Experimental Colitis by Modulating Inflammation and Regulating the Gut Microbiota
Source: Nutrients. 2026 Jul 20;18(14):2369. doi: 10.3390/nu18142369 (PMC13415326; doi:10.3390/nu18142369)
Supplement: Supplementary file 1 [file nutrients-18-02369-s001.zip › nutrients-4407985-supplementary.pdf]

Supplemental Information

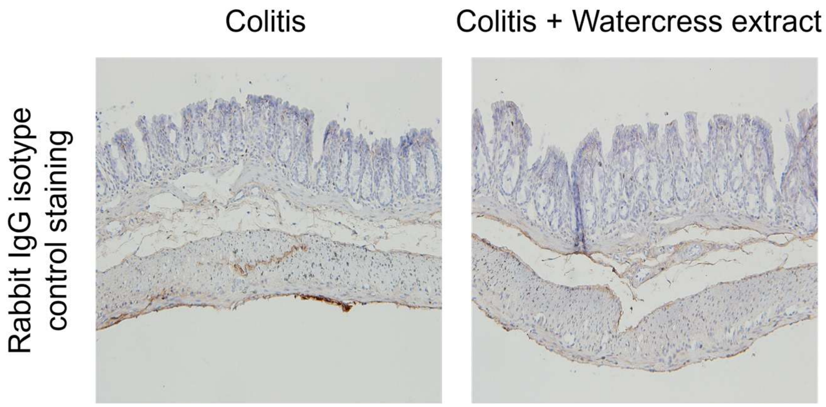

Figure S1. Negative control staining for immunohistochemistry. Representative images of rabbit IgG isotype control staining in colonic tissues from DSS-induced colitis mice with or without watercress extract supplementation (magnification 200X).

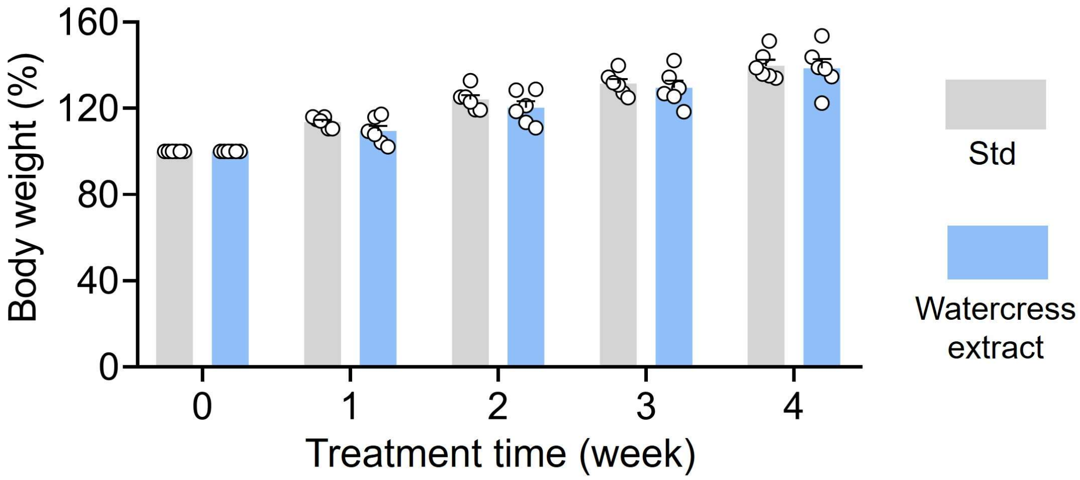

Figure S2. Watercress extract supplementation did not affect body weight during the dietary pretreatment period. Six-week-old male C57BL/6 mice were fed either a standard AIN-93G diet (Std) or a modified AIN-93G diet supplemented with 0.5% (w/w) watercress extract for 4 weeks. Data are presented as mean ± SEM. n = 6 mice per group. No significant difference was detected between groups.

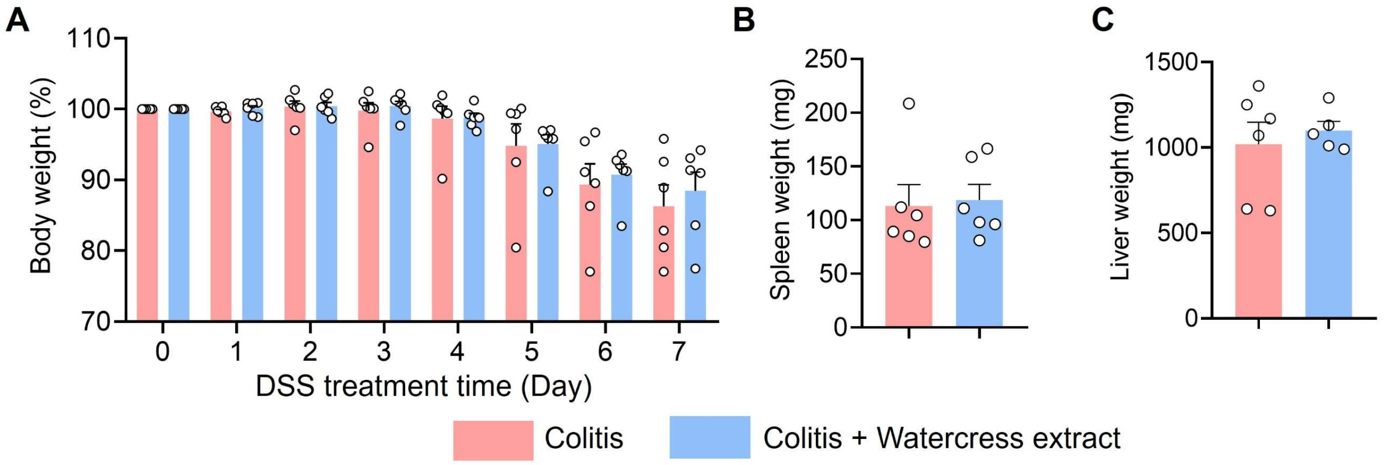

Figure S3. Watercress extract supplementation did not influence overall body weight loss, spleen weight, or liver weight during DSS-induced colitis. (**A**) Body weight changes during DSS treatment. (**B**) Spleen weight and (**C**) liver weight at the experimental endpoint. Data are presented as mean ± SEM. n = 6 mice per group. No significant differences were detected between groups.

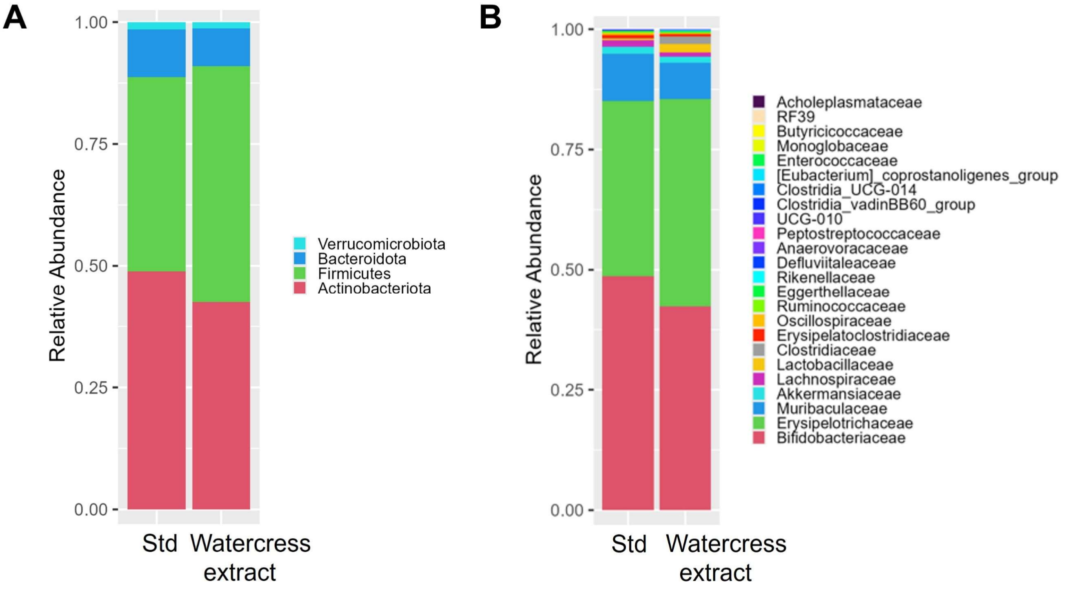

Figure S4. Watercress extract supplementation alters gut microbial composition at phylum and family levels. Fecal samples collected from mice fed either a standard AIN-93G diet (Std) or a watercress extract-supplemented diet for 4 weeks prior to DSS administration were subjected to *16S rRNA* gene amplicon sequencing analysis. Relative taxonomic composition of the fecal microbiota is shown at (**A**) phylum level and (**B**) family level.

Table S1. Nutritional composition of the watercress extract powder

Nutrient	Value
Dry matter (%)	93.89 ± 0.39
Crude protein (%)	5.64 ± 0.06
Crude fat (%)	3.43 ± 0.23
Ash (%)	5.17 ± 0.04
Calcium (%)	0.46 ± 0.06
Phosphorus (%)	0.07 ± 0.01
Gross energy (kcal/g)	3.70 ± 0.002

** All analyses were performed in duplicate, and results are reported on an as-is basis.*

*** The nutritional composition of the watercress extract powder was determined using standard analytical procedures. Dry matter content of watercress extract sample was determined by drying samples at 105 °C in a forced-air drying oven (Precision Scientific Co., Chicago, IL, USA; AOAC method 934.01) until a constant weight was achieved. Crude protein was analyzed by determining nitrogen concentration using the combustion method (TruMac® N; LECO Corp., St. Joseph, MI, USA; AOAC method 990.03) and calculating crude protein as N × 6.25. Crude fat was determined by ether extraction (AOAC method 920.39), and ash content was measured by combustion in a muffle furnace at 550 °C (AOAC method 942.05). Gross energy was determined using an isoperibol bomb calorimeter (Parr 6200; Parr Instrument Co., Moline, IL, USA).*

**** Neutral detergent fiber (NDF) and acid detergent fiber (ADF) concentrations were determined using the filter bag technique with an ANKOM A200 Fiber Analyzer (ANKOM Technology, Macedon, NY, USA) according to the manufacturer's procedures. Both NDF and ADF concentrations were below the method detection limit and therefore could not be quantified.*

Table S2. Sequences of primers in qPCR analysis

Mouse primer information		
**Gene**	**Forward primer**	**Reverse primer**
Gapdh	AGGTCGGTGTGAACGGATTTG	TGTAGACCATGTAGTTGAGGTCA
Actb	GGCTGTATTCCCCTCCATCG	CCAGTTGGTAACAATGCCATGT
Cd45	GTTTTCGCTACATGACTGCACA	AGGTTGTCCAACTGACATCTTTC
Tnf-α	CCCTCACACTCAGATCATCTTCT	GCTACGACGTGGGCTACAG
Ccl-3	TTCTCTGTACCATGACACTCTGC	CGTGGAATCTTCCGGCTGTAG
Muc2	ATGCCCACCTCCTCAAAGAC	GTAGTTTCCGTTGGAACAGTGAA
Claudin-3	ACCAACTGCGTACAAGACGAG	CAGAGCCGCCAACAGGAAA
Zo-1	GGGGCCTACACTGATCAAGA	TGGAGATGAGGCTTCTGCTT
Mcp-1	TTAAAAACCTGGATCGGAACCAA	GCATTAGCTTCAGATTTACGGGT
Il-1β	GCAACTGTTCCTGAACTCAACT	ATCTTTTGGGGTCCGTCAACT
Ccl-4	TTCCTGCTGTTTCTCTTACACCT	CTGTCTGCCTCTTTTGGTCAG
Tgf-β	TTCTCTGTACCATGACACTCTGC	CGTGGAATCTTCCGGCTGTAG
Catalase	AGCGACCAGATGAAGCAGTG	TCCGCTCTCTGTCAAAGTGTG

Table S3. Effects of watercress extract on the composition of gut microbiota at genus levels (results are expressed as relative abundance)

	Standard diet		Watercress extract diet		
**Genus**	**Average**	**SEM**	**Average**	**SEM**	***P* value**
Acetatifactor	0.0000459	0.0000398	0.0000721	0.0000199	0.575
Adlercreutzia	0.0000000	0.0000000	0.0000474	0.0000148	**0.015**
Akkermansia	0.0150307	0.0037722	0.0132568	0.0030655	0.723
Alistipes	0.0004689	0.0003055	0.0021810	0.0008899	0.117
Anaeroplasma	0.0000263	0.0000202	0.0000506	0.0000339	0.554
Anaerotruncus	0.0004494	0.0000784	0.0001273	0.0000489	**0.006**
ASF356	0.0001163	0.0000291	0.0000504	0.0000093	0.075
Bifidobacterium	0.4864384	0.0722417	0.4232057	0.0545093	0.502
Blautia	0.0004294	0.0002431	0.0000830	0.0000598	0.219
Butyricicoccus	0.0000257	0.0000123	0.0000000	0.0000000	0.090
Clostridia	0.0000007	0.0000004	0.0000018	0.0000011	0.407
Clostridia_UCG-014	0.0002745	0.0001537	0.0000672	0.0000290	0.239
Clostridia_vadinBB60_group	0.0001158	0.0000372	0.0003482	0.0001476	0.181
Clostridium_sensu_stricto_1	0.0001463	0.0000789	0.0158143	0.0101109	0.182
Colidextribacter	0.0010132	0.0003112	0.0005378	0.0001050	0.197
Coprococcus	0.0000032	0.0000032	0.0000000	0.0000000	0.363
Defluviitaleaceae_UCG-011	0.0006617	0.0001806	0.0003582	0.0000631	0.162
Dubosiella	0.0679455	0.0100187	0.0841152	0.0200814	0.493
Enterococcus	0.0001252	0.0000386	0.0000373	0.0000075	**0.015**
Enterorhabdus	0.0023736	0.0003152	0.0015226	0.0001423	**0.034**
Erysipelatoclostridiaceae	0.0006104	0.0000589	0.0004255	0.0001891	0.387
Erysipelatoclostridium	0.0062977	0.0017755	0.0040328	0.0025815	0.488
Erysipelotrichaceae	0.0000245	0.0000076	0.0000175	0.0000059	0.484
Faecalibaculum	0.2962494	0.0831242	0.3474567	0.0742452	0.656
Family_XIII_AD3011_group	0.0000242	0.0000056	0.0000212	0.0000062	0.727
Family_XIII_UCG-001	0.0000723	0.0000161	0.0000497	0.0000114	0.281
GCA-900066575	0.0013830	0.0005695	0.0006627	0.0001670	0.272
Harryflintia	0.0000254	0.0000145	0.0000063	0.0000037	0.253
Incertae_Sedis	0.0006029	0.0001497	0.0004619	0.0001281	0.491
Intestinimonas	0.0002419	0.0000891	0.0001153	0.0000243	0.222
Lachnoclostridium	0.0011703	0.0004964	0.0004671	0.0001275	0.222
Lachnospiraceae_FCS020_group	0.0001180	0.0000294	0.0000702	0.0000133	0.183
Lachnospiraceae_NK4A136_group	0.0041786	0.0016848	0.0029755	0.0010974	0.565
Lachnospiraceae_UCG-004	0.0000064	0.0000064	0.0000000	0.0000000	0.363
Lachnospiraceae_UCG-006	0.0002593	0.0000942	0.0002010	0.0000461	0.595
Lachnospiraceae_UCG-010	0.0000018	0.0000018	0.0000000	0.0000000	0.363
Lactobacillus	0.0032442	0.0014123	0.0177688	0.0071107	0.097
Marvinbryantia	0.0000158	0.0000077	0.0000621	0.0000361	0.261
Monoglobus	0.0000401	0.0000088	0.0000851	0.0000164	**0.036**

Muribaculaceae	0.0978192	0.0087071	0.0752713	0.0088542	0.099
NK4A214_group	0.0000434	0.0000280	0.0000110	0.0000110	0.319
Oscillibacter	0.0002550	0.0001150	0.0002153	0.0000698	0.775
Paeniclostridium	0.0000000	0.0000000	0.0000005	0.0000005	0.363
Paludicola	0.0000006	0.0000006	0.0000008	0.0000008	0.819
Parvibacter	0.0000045	0.0000029	0.0000308	0.0000308	0.434
Pasteurella	0.0000000	0.0000000	0.0000006	0.0000006	0.363
RF39	0.0000399	0.0000139	0.0000609	0.0000248	0.481
Romboutsia	0.0004390	0.0003376	0.0003266	0.0001766	0.776
Roseburia	0.0005600	0.0001227	0.0003002	0.0001227	0.165
Staphylococcus	0.0000300	0.0000140	0.0000053	0.0000034	0.141
Tuzzerella	0.0002076	0.0000606	0.0001366	0.0000298	0.327
UCG-005	0.0002644	0.0001184	0.0000499	0.0000499	0.141
UCG-009	0.0000535	0.0000160	0.0000286	0.0000094	0.215
UCG-010	0.0002901	0.0000260	0.0002174	0.0000285	0.089
Unclassified Erysipelotrichaceae	0.0000060	0.0000060	0.0000362	0.0000362	0.445
Unclassified Lachnospiraceae	0.0016946	0.0003762	0.0016234	0.0005258	0.915
Unclassified Oscillospiraceae	0.0011049	0.0003102	0.0006645	0.0001187	0.230
Unclassified Oscillospirales	0.0000116	0.0000029	0.0000055	0.0000014	0.104
Unclassified Ruminococcaceae	0.0005646	0.0001641	0.0006881	0.0001029	0.541
Uncultured Erysipelotrichaceae	0.0000067	0.0000052	0.0000127	0.0000127	0.675
Uncultured Lachnospiraceae	0.0036091	0.0013856	0.0015498	0.0002411	0.200
Uncultured Oscillospiraceae	0.0013790	0.0002512	0.0007770	0.0001304	**0.041**
Uncultured Peptococcaceae	0.0000457	0.0000062	0.0000250	0.0000028	**0.012**
Uncultured Ruminococcaceae	0.0007973	0.0001739	0.0004744	0.0000935	0.142
[Eubacterium]_brachy_group	0.0000999	0.0000244	0.0000679	0.0000151	0.297
[Eubacterium]_coprostanoligenes_group	0.0000000	0.0000000	0.0003238	0.0003238	0.363
[Eubacterium]_nodatum_group	0.0003700	0.0001488	0.0002701	0.0000305	0.538
[Eubacterium]_ventriosum_group	0.0000437	0.0000151	0.0000235	0.0000076	0.271
[Eubacterium]_xylanophilum_group	0.0000018	0.0000018	0.0000442	0.0000199	0.086
